# Enhanced Recovery after Pelvic Organ Prolapse Surgery

**DOI:** 10.3390/jcm12185911

**Published:** 2023-09-12

**Authors:** Caroline Tresch, Marine Lallemant, Rajeev Ramanah

**Affiliations:** 1 Service de Gynécologie-Obstétrique, Université de Franche-Comté, CHU de Besançon, 25000 Besançon, France; mlallemant@chu-besancon.fr; 2 Service de Gynécologie, Université de Franche-Comté, CHU de Besançon, 25000 Besançon, France; rajeev.ramanah@univ-fcomte.fr

**Keywords:** pelvic organ prolapse, prolapse, vaginal surgery, vulvar surgery, urogynecologic surgery, sacrocolpopexy, enhanced recovery after surgery

## Abstract

The objective of this study was to review on the influence of enhanced rehabilitation in pelvic organ prolapse surgery outcomes, specifically focusing on length of hospital stay, hospital costs, pain, morbidity, and patient satisfaction. Following the PRISMA model and using PubMed as a source, eight articles pertaining to prolapse surgery and two articles concerning vaginal hysterectomies were selected, all published between 2014 and 2021. These studies revealed no significant difference in terms of operating time, intra- and post-operative complications, intra-operative blood loss and post-operative pain scores before and after the introduction of the ERAS program. Only one study noted a difference in readmission rates. There was, however, a noticeable decrease in intra-operative and post-operative intravenous intakes, opioid administration, length of stay, and overall hospital costs with the adoption of ERAS. Additionally, with ERAS, patients were able to mobilize more rapidly, and overall patient satisfaction significantly improved.

## 1. Introduction

Enhanced recovery after surgery (ERAS) is a multimodal therapeutic approach introduced in the 1990s by Professor Henrik Kehlet and his team from the University of Copenhagen. This method is grounded on four primary elements: optimized pre-operative information and education, intra-operative fluid management, effective analgesia, and prompt post-operative mobilization paired with early refeeding [1]. It employs a comprehensive and coordinated multidisciplinary approach, engaging the entire healthcare team, including surgeons, anesthetists, nurses, physiotherapists, dieticians, and primary care physicians, throughout all phases of care: pre-operative, peri-operative, and post-operative. Furthermore, ERAS provides holistic patient care, addressing physical, emotional, economic, and social health dimensions [2]. The patient is thus a full actor of their care. Thus, the patient becomes an active participant in their treatment. The surgical methods adopted prioritize minimally invasive techniques and limit the use of drains. Anesthetic protocols are tailored to include multimodal analgesia, minimize opioid usage, and prevent post-operative nausea and vomiting.

While initially developed for colorectal [3], the goals of the ERAS program are to diminish psychological stress prior to surgery, reduce metabolic disturbances, promote improved recovery, shorten hospital stays without an uptick in post-operative complications, and enhance the quality of care and patient satisfaction [4]. 

Over the past decade, ERAS has seen substantial growth and has been implemented in over 21 countries [1]. It has expanded to encompass various surgical specialties, including digestive surgery, urology, cardiovascular and thoracic surgery, orthopedics, and obstetrics, and gynecology. Using the colorectal surgery model as a blueprint, ERAS guidelines were released for vulvar and vaginal surgery in July 2020 [5] and for urogynecologic surgery in 2022 [6]. The underlying premise of our study was to investigate if the application of ERAS in pelvic organ prolapse surgery could potentially reduce both the length of hospital stays and costs, and enhance patient satisfaction, without escalating pain or the incidence of post-operative complications and readmissions.

The main objective of this research was to conduct a literature review examining the impact of ERAS on pelvic organ prolapse surgery, considering factors such as hospital stay duration, associated costs, pain, morbidity, and patient satisfaction.

## 2. Materials and Methods

Following the PRISMA guidelines, we conducted a search on PubMed using the following MESH keywords in either the title or abstract: “pelvic organ prolapse”, “prolapse”, “vaginal surgery”, “vulvar surgery”, “urogynecologic surgery”, “sacrocolpopexy”, “enhanced recovery after surgery”, and “ERAS”. We excluded clinical practice guidelines and literature review articles from our search. Articles that did not align with the objectives of our literature review were also excluded. Only those articles that compared the length of hospital stay, hospital costs, pain, morbidity, and patient satisfaction before and after the implementation of an enhanced rehabilitation protocol following functional pelvic surgery were considered for our study.

## 3. Results

**Figure 1:**
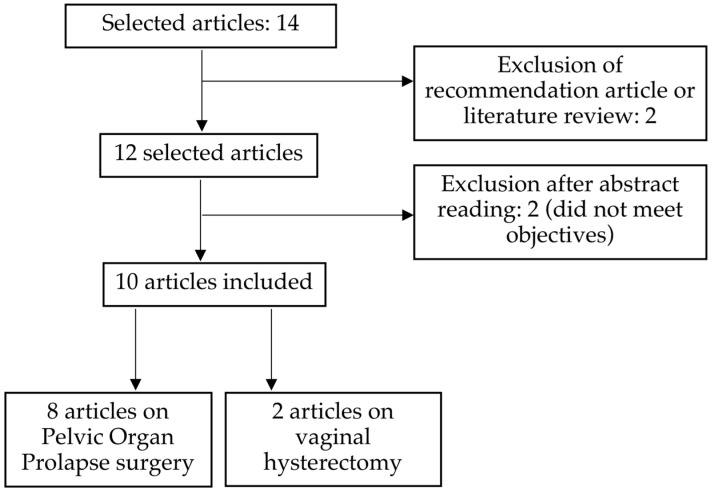
Flow chart.

A total of 14 articles were identified on PubMed where the keywords were included either in the title or the abstract. Of these 14 articles, one was excluded after the abstract was reviewed because it focused on ERAS recommendations specific to pelvic floor reconstructive surgery. Another article was discarded as its focus was on recommendations for sacrocolpopexy. Two more articles were omitted because they were not aligned with the objectives of our study. Eventually, eight articles related to pelvic organ prolapse surgery and two articles concerning vaginal hysterectomies, all published between 2014 and 2021, were chosen for review. The selection process is depicted in Figure 1.

The characteristics of studies focusing on pelvic organ prolapse and vaginal hysterectomies are detailed in Table 1. No studies were found that dealt with perineal surgery. These studies aimed to evaluate the impact of an enhanced rehabilitation program following pelvic floor reconstructive surgery, particularly regarding the length of hospital stay, the rate of discharge on day 0, pain, post-operative complications, readmission rates, and economic implications. One study specifically explored feedback from patients treated within the ERAS framework. The surgical procedures and the objectives of each study are outlined in Table 2.

Patient characteristics are summarized in Table 3. The average age of the patients was 60 years. The majority were slightly overweight with an average BMI (Body Mass Index) of approximately 27–28 kg/m². Most patients had an ASA score of 2, indicating a mild systemic disease. In the study conducted by Carter-Brooks et al., 66.3% of the patients had a history of abdominal surgery [7], whereas around 25% had a history of laparotomy in other studies [8,9,10]. Among the two articles discussing vaginal hysterectomy, prolapse was the primary reason for hysterectomy in 34.5% of cases in Relph et al.’s study [9] and in 35% of cases in Yoong et al.’s study [10]. The stage of the prolapse was not specified. The average parity was 2.75 in Relph et al.’s study [9] and 2 in Yoong et al.’s study [10].

**Table 1:** Characteristics of studies of pelvic organ prolapse surgery and vaginal hysterectomy in enhanced rehabilitation.

	Type of Study	Time Period	Number of Patients
Carter-Brooks [7] USA 2018	Observational Retrospective and prospective Single-center	January 2016– June 2016	137 not ERAS 121 ERAS
Evans [8] USA 2021	Observational Retrospective Single-center	January 2017– June 2019	83 not ERAS 83 ERAS
Gong [11] China 2020	Observational Retrospective Single-center	August 2015– November 2017	85 not ERAS 92 ERAS
Abrao Trad [12] USA 2020	Observational Retrospective Single-center	April 2009– November 2017	128 not ERAS 83 ERAS
Trowbridge [13] USA 2018	Observational Retrospective and prospective Single-center	January 2014– February 2016	76 not ERAS 118 ERAS
Evans [14] USA 2020	Observational Retrospective Single-center	December 2018–February 2019	14 ERAS (7 outputs at day 0 and 7 outputs at day 1)
Pan [15] India 2021	Observational Retrospective and prospective Single-center	December 2013–February 2020	169 not ERAS 89 ERAS
Carter-Brooks [16] USA 2021	Observational Retrospective Single-center	January 2016– October 2017	210 not ERAS 172 ERAS (3 groups: <61 years, 61 to 75 years, >75 years)
Relph [9] England 2014	Observational Retrospective and prospective Single-center	November 2010–February 2012	45 not ERAS 45 ERAS
Yoong [10] England 2014	Observational Single-center	November 2009–March 2012	50 not ERAS 50 ERAS

**Table 2:** Type of surgery and aim of each study.

	Type of Surgery	Aim of the Study
Carter- Brooks [7]	Laparoscopic or robot-assisted prolapse surgery: 43.4% Vaginal prolapse surgery: 22.5% Colpocleisis: 23.6% ±Hysterectomy: 57.4%	To evaluate whether the implementation of ERAS resulted in a reduction in hospital length of stay
Evans [8]	Laparoscopic sacrocolpopexy (44%) and robotic-assisted sacrocolpopexy (56%) with: - Hysterectomy: 46% - Adnexal surgery: 22.5% - Sub-urethral sling: 32% - Posterior wall repair: 74.5% Other: 20.5%	To assess whether ERAS was associated with a higher rate of day 0 discharge after laparoscopic (±robotic assisted) sacrocolpopexy and to describe the safety and feasibility of day 0 discharge after this type of procedure
Gong [11]	Pelvic organ prolapse surgery with vaginal mesh	To study the impact of ERAS on intra-operative outcomes after pelvic organ prolapse surgery with vaginal mesh
Abrao Trad [12]	Sacrocolpopexy	To evaluate the impact of ERAS on drug use, length of hospital stay, costs, and morbidity
Trowbridge [13]	Pelvic organ prolapse surgery: - Reconstructive: 98.7% without ERAS vs. 91.5% with ERAS: Prolapse treatment with vaginal hysterectomy: 84.2% vs. 58.5% Prolapse treatment by robotic surgery 7.9% vs. 22.9% Other vaginal reconstruction (prolapse treatment without hysterectomy, with or without suspension of the vaginal apex, TOT/TVT placement, mesh excision): 6.6% vs. 10.2% - Obliterative (colpocleisis): 1.3% vs. 8.5%	To evaluate outcomes after an ERAS program for minimally invasive or vaginal pelvic surgery
Evans [14]	Sacrocolpopexy by laparoscopy or robot-assisted laparoscopy ±concomitant surgery	To describe patient experiences with an enhanced recovery protocol after minimally invasive sacrocolpopexy
Pan [15]	Sacrocolpopexy by laparoscopy	To determine whether an ERAS program improved the perception of post-operative recovery after laparoscopic sacrocolpopexy
Carter- Brooks [16]	Laparoscopic sacrocolpopexy: 42.7%. Vaginal apex suspension (sacrospinous ligament or utero- sacral ligament fixation): 30.3% Colpocleisis: 27% ±Hysterectomy: 56.3%	To evaluate discharges at day 0, opioid administration, pain scores, and differences in complications in three age categories (<61 years, 61 to 75 years, >75 years), before and after implementation of ERAS.
Relph [9]	Vaginal hysterectomy	To explore the cost savings after implementation of an ERAS program for vaginal hysterectomy
Yoong [10]	Vaginal hysterectomy	To evaluate the impact of ERAS on patients after vaginal hysterectomy for benign indications.

**Table 3:** Patient characteristics.

	Age (Years)	BMI (kg/m^2^)	ASA Score	Stage of Prolapse	History of Laparotomy
Carter-Brooks [7]	65	28.2	NA	0: 4.3% 1: 0.4% 2: 20.2% 3: 65.1% 4: 10.1%	NA
Evans [8]	64.3	26.9	1: 5.5% 2: 73% 3: 21% 4: 0.5%	1: 3.5% 2: 35.5% 3: 50% 4: 11%	26%
Gong [11]	67	28.9	NA	2: 4.5% 3: 72.9% 4: 22.6%	NA
Abrao Trad [12]	NA	NA	NA	NA	NA
Trowbridge [13]	58.1	28.3	1: 9% 2: 75% 3: 16%	NA	NA
Evans [14]	65.5	26.8	2: 71.4% 3: 28.6%	2: 42.9% 3: 57.1%	NA
Pan [15]	62.9	28.1	NA	NA	NA
Carter-Brooks [16]	66	27.8	NA	2: 16.8% 3: 70.7% 4: 12.5%	NA
Relph [9]	53	27.85	NA	NA	26.6%
Yoong [10]	50	NA	1 to 3: 96%	NA	24%

BMI: Body Mass Index; ASA: American Society of Anesthesiologists; NA: not available.

Upon analyzing the various articles, five key areas of interest emerged: intra-operative and post-operative effects of ERAS, post-operative complications, cost implications, and patient satisfaction.

### 3.1. Contributions of ERAS in the Intra-Operative

#### 3.1.1. Operating Time

The studies found no significant difference in operating time before and after the implementation of the ERAS program [7,8,10,11,13,14,15] (Table 4). In the study by Gong et al., while the total operating room time did show a statistically significant reduction, it was not clinically meaningful, decreasing from 98 min to 93 min (*p* = 0.003) [11].

#### 3.1.2. Intra-Operative Complications

There was no discernible difference observed regarding intra-operative complications [7,8].

#### 3.1.3. Intra-Operative Blood Loss

There was no observed difference in intra-operative blood loss when comparing before and after the implementation of the ERAS [7,8,10,11,13,14] except in Pan et al.’s study [15], although this result was not clinically significant (Table 4).

#### 3.1.4. Intra-Operative Intravenous Intakes

Trowbridge et al. reported a reduction in intra-operative intravenous intakes from 1403 mL to 690 mL (*p* < 0.0001). During hospitalization, intakes dropped from 2694 mL to 1473 mL (*p* < 0.0001), with total intakes decreasing from 2.7 L to 1.5 L (*p* < 0.0001) [13]. However, in Gong et al.’s study, although intra-operative intravenous intakes also declined, from 725.9 to 678.5 mL, this reduction was not statistically significant (*p* = 0.119) [11].

#### 3.1.5. Intra-Operative Morphine Rate

In Trowbridge et al.’s research, the intra-operative morphine dose saw a marked decline from 16.02 mg to 1.92 mg (*p* < 0.0001) [13]. Carter-Brooks et al. found a 79% reduction in perioperative opioid administration with ERAS, moving from 87.1 mg to 18.4 mg oral morphine equivalent (*p* < 0.0001) [16].

### 3.2. Benefits of ERAS in Post-Operative Care

#### 3.2.1. Length of Stay

Length of Stay: All studies reported a significant decrease in the length of hospital stay (Table 4). Notably, Carter-Brooks et al. observed an increase in day 0 discharges, from 25.9% to 91.7% (*p* < 0.001) [7], and in Evan et al.’s study, from 12% to 40% (*p* < 0.01) [8]. In Abrao Trad et al.’s study, more than one-day stays dropped from 98.4% to 91.6% (*p* < 0.05) [12].

#### 3.2.2. Post-Operative Opioids

Trowbridge et al. noted a minor reduction in post-operative morphine dose from 21.4 mg to 17.6 mg (*p* = 0.1), but a significant decline in the average in-hospital morphine dose from 37.4 mg to 19.4 mg (*p* < 0.01) [13]. In the study by Abrao Trad et al., the use of patient-controlled analgesia post-operatively decreased from 83.6% to 8.4% on day 0 and from 85.8% to 12.0% on day 1. Additionally, opioid use in the recovery room declined from 72.2% to 61.4%. On day 0, it decreased from 89.1% to 72.3%, and on day 1, it reduced from 93.8% to 89.2% [12].

#### 3.2.3. Pain

There were no significant differences in post-operative pain scores across several studies. Carter-Brooks et al. reported consistent pain scores in the recovery room (*p* = 0.762 [16]. Similarly, Trowbridge et al. found no difference in pain scores on day 0 (4.49 vs. 4.29, *p* = 0.545), day 1 (3.40 vs. 3.36, *p* = 0.904), or in the maximum pain reported by patients (7.39 vs. 7.37, *p* = 0.95) [13]. Yoong et al. also reported consistent pain scores at discharge (2.7 vs. 2.5) [10]. Notably, in Carter-Brooks et al.’s study, 86.7% of patients rated their pain control as very good or excellent [7].

#### 3.2.4. Mobilization, Walking, and Recovery

Under the ERAS program, patients mobilized faster compared to a conventional approach. In the study by Cong et al., patients took their first walk after 23.56 h under ERAS, in contrast to 40.6 h pre-ERAS (*p* < 0.001) [11]. Trowbridge et al.’s research found that patients in the enhanced rehabilitation group began ambulating more frequently by day 0 (73.7% compared to 17.1%, *p* < 0.01) and by day 1 (72.9% compared to 34.2%, *p* < 0.01) [13]. In the study by Pan et al., patients in the ERAS group reported a significantly higher mean post-discharge surgical recovery (PSR) scale 13 score than before ERAS, increasing from 62.3 to 68.4 (95% CI, 2.04–10.16). Pan et al. reported that patients under ERAS indicated a notably improved average post-discharge surgical recovery (PSR) scale 13 score, which rose from 62.3 to 68.4 (95% CI, 2.04–10.16) [15].

### 3.3. Post-Operative Complications

#### 3.3.1. Readmission

There was no significant difference in readmission rates across the studies, with the exception of Carter-Brooks et al.’s study. In this study, the readmission rate increased from 1.5% to 6.7% [7,9,10,12,13] (Table 4). In the same study, patients from the pre-ERAS group were readmitted due to myocardial infarction and chest pain, while those in the ERAS group were readmitted for reasons such as weakness, chest pain, hyponatremia, wound complications, nausea/ileus, and ureteral obstruction [7].

#### 3.3.2. Post-Operative Complications within 30 Days and at One Year after Surgery

There was no significant difference in post-operative complications at 30 days [7,8,10,12,13,16] and at one year [11]. The details can be found in Table 5.

### 3.4. Hospital Cost

Most studies observed a decrease in total hospital costs, except for the study by Trowbridge et al. In their study, the average hospital costs at 30 days showed no significant difference between the two groups. Similarly, there were no significant differences in the average indirect hospital costs (USD 3631 vs. USD 3407, *p* = 0.445) or in the average direct hospital costs (USD 4277 vs. USD 4571, *p* = 0.375) [13]. In Relph et al.’s study, a reduction of 19.4 h in the length of stay led to savings of GBP 176 (equivalent to EUR 210) or GBP 9.08 (EUR 10.8) per hour. However, the actual amount saved was GBP 165 (or EUR 196) [9] (Table 4).

### 3.5. Patient Satisfaction

In the study by Carter-Brooks et al., 89.6% of patients rated the preparation for surgery as very good or excellent, and 93.5% presented the same rating for the overall management. Satisfaction was assessed using a phone-administered questionnaire the day after discharge [7]. In the study by Trowbridge et al., patient satisfaction significantly improved: 90.9% of patients recommended their department with ERAS, compared to 71.4% before its implementation (*p* = 0.0005). Satisfaction was gauged using the Hospital Consumer Assessment of Healthcare Providers and Systems (HCAHPS) voluntary patient surveys, which were administered post-discharge [13]. In the study by Yoong et al., the median satisfaction score was 8/10 at four weeks post-surgery. A score of 9/10 was obtained in 65% of cases. However, 7% of patients rated the ERAS program as 1/10, and one patient felt pressured to discharge early. Satisfaction was gauged using questionnaires (based on a 10 cm VAS) that were mailed to patients and completed four weeks after discharge [10].

In the study by Evans et al., which focused on patient feedback, seven patients were discharged on day 0 and another seven were discharged on day 1. The findings generally highlighted positive feedback regarding enhanced rehabilitation after minimally invasive sacrocolpopexy. Satisfaction was gauged using telephone interviews conducted between 2 and 6 weeks post-discharge. Although an interview guide was used, patients were encouraged to narrate their experiences in their own words. The majority felt well-informed about the procedure and post-operative management, leading to reduced anxiety upon hospital admission. They expressed satisfaction with their swift return home and appreciated the familiarity and comfort of their environment. Many felt confident in their ability to assess their health and to contact a healthcare professional post-discharge if needed. Several emphasized the importance of family and friend support during their return home. On the other hand, one patient felt that the enhanced rehabilitation protocol prioritized economic considerations over patient recovery. Patients who favored discharge on day 0 commonly cited reasons such as family support at home, good personal health, and the ability to quickly access medical care. Those preferring to stay the first post-operative night in the hospital raised concerns about potential difficulties accessing care from home, worries about their health status, or cited their advanced age. Regardless of preference, the option to stay overnight in the hospital was positively received by all. Overall, patients believed that ERAS ensured a more seamless transition and continuity of care upon discharge.

The negative point reported by patients was being discharged with a urinary catheter. Some of those discharged with an indwelling urinary catheter felt the discharge process was hastened and expressed a desire to have stayed in the hospital for an additional night. Despite being informed pre-operatively about the potential for this outcome, a few voiced dissatisfaction and distress regarding the device. Their concerns were not linked to complications arising from the urinary catheter itself, but rather stemmed from the fear of needing it permanently [14].

## 4. Discussion

To the best of our knowledge, this study is unique in accounting for the current understanding of the influence of enhanced rehabilitation in pelvic organ prolapse surgery.

During surgery, the ERAS program showed no significant impact on operative time, intra-operative complications, or blood loss. Such an outcome is unsurprising given that each study compared the same surgical procedure, both with and without the ERAS program. Consequently, there is no reason to believe that ERAS would affect the surgical procedure or influence blood loss. However, intra-operative morphine consumption was reduced. Several studies have indicated that ERAS contributes to a decrease in both intra- and post-operative morphine doses in gynecology. These reductions vary from 20 to 64% intra-operatively depending on the study, and can reach up to 84% post-operatively [17,18,19,20,21,22,23,24]. Intra-operative intravenous fluid consumption was also significantly decreased by 700 mL, and total fluid consumption was reduced by more than a liter. This aligns with the ERAS program’s recommendation for maintaining euvolemia [5].

Post-operatively, every study in our literature review reported a significant reduction in the length of stay, coupled with an increased proportion of discharges on the day of the procedure. This outcome mirrors the findings from the majority of studies focusing on ERAS in gynecology, which consistently demonstrate a reduction in hospital stays [7,19,20,21,22,23,24,25,26,27,28,29]. Numerous studies have indicated that same-day discharges are safe, following vaginal hysterectomy with pelvic floor reconstruction [30] and after minimally invasive or robotic-assisted pelvic floor reconstruction [31,32,33].

Opioid consumption post-operatively declined without any notable difference in pain scores. This aligns with several studies examining ERAS in the context of pelvic organ prolapse surgery [18,19,20,22,24,27]. Furthermore, under the ERAS protocol, patients exhibited quicker mobilization. Notably, those in the ERAS group recorded a significantly higher mean surgical recovery score post-discharge compared to pre-ERAS evaluations.

Overall, no discernible difference was found in terms of readmissions (except in the study by Carter-Brooks et al. [7]), which indicated readmission rates between 3.4% and 6%. The majority of studies on ERAS in pelvic organ prolapse surgery also did not observe any difference in readmission rates, irrespective of whether they recorded a reduction in the initial hospital stay. Nevertheless, readmission rates exhibited significant variability across studies, ranging from 2.3% to 25% [17,18,19,20,21,22,23,24,25,26,27,28,29]. Likewise, no discernible difference emerged concerning post-operative complications at both 30 days and one year. However, the complication rate varied considerably between studies, depending on the criteria employed, with rates ranging from 1% to 35.5%. Comparing morbidity across multiple publications proves challenging due to the different parameters assessed in each study. Morbidity criteria’s severity can also vary, thereby explaining the substantial differences observed. While some authors provided an overall morbidity based on multiple criteria, others analyzed morbidity on a point-by-point basis. Abrao Trad et al. did not delineate the criteria used for evaluating morbidity [12]. Carter-Brooks et al. ambiguously defined morbidity as “any aberration in the normal recovery” [16]. Yoong et al. merely addressed emergency room visits without delving into the broader topic of morbidity [10]. Such disparities among publications could influence result interpretation and comparability. Employing the Clavien–Dindo classification might address this issue, yet it was not utilized in the studies included in our literature review. Ultimately, one must ponder the merits of vaginal versus laparoscopic approaches, an aspect not explored in the chosen studies. The surgical treatment option for patients with pelvic organ prolapse might positively influence patients’ quality of life. Illiano et al. reported a higher incidence of urinary tract infections with a vaginal approach compared to the laparoscopic method, suggesting the latter might be more advantageous [34].

The majority of studies observed a reduction in overall hospital stay costs. Analogously, in gynecological oncology, a Canadian study indicated that an enhancement in the ERAS program’s compliance from 56% to 77% correlated with a 31.4% decline in the length of stay and a net savings of CAD 952 per patient [25]. In colorectal surgery, the program further yielded notable cost reductions: an average saving of USD 2240 (EUR 1885) per patient, as demonstrated by a Massachusetts study [35].

Furthermore, patient satisfaction generally ranged from good to very good. An exception was noted among patients discharged with an indwelling urinary catheter, underscoring the significance of comprehensive pre- and post-operative education to bolster patient satisfaction. It is pertinent to mention that ERAS guidelines strongly advocate for the prompt removal of the urinary catheter [6]. This observation aligns with other studies, which have reported enhanced satisfaction among gynecological patients managed via the ERAS protocol [21,22,27,36].

Our literature review is subject to several limitations. Primarily, limited studies address ERAS in pelvic floor surgery, and those that exist are solely retrospective, single-center studies with a low level of evidence, introducing potential reporting bias. A significant drawback of our analysis is the heterogeneity of the studies chosen. They vary in the type of surgery, with two focused on vaginal hysterectomy, one on prolapse surgery employing a vaginal mesh, five on sacrocolpopexy, and two on diverse prolapse surgery methods (e.g., laparoscopic or robotic-assisted sacrocolpopexy, vaginal prolapse surgery, and colpocleisis). Additionally, some studies featured complementary procedures (such as the insertion of sub-urethral transvaginal or transobturator tape, salpingo-oophorectomy, or mesh excision), further complicating result interpretation. The surgical techniques often lacked detailed descriptions. Concerning the prolapse stage, it remains ambiguous whether authors used the Baden–Walker or the POP-Q classification, potentially introducing classification and interpretation biases. Due to these discrepancies, the studies are challenging to compare, making a meta-analysis infeasible. Future research should prioritize prospective studies with larger cohorts to enhance data analysis quality. Another challenge is the absence of a universal ERAS protocol standard, with each team devising its protocol. Nevertheless, the congruence in overall outcomes across studies bolsters the external validity of the various investigations.

## 5. Conclusions

Based on our literature review, implementing ERAS in urogynecology led to reductions in both intra-operative and post-operative intravenous fluids and morphine doses, without any disparity in pain scores. With ERAS adoption, patients experienced a quicker mobilization and notably higher mean surgical recovery scores post-discharge compared to pre-ERAS scenarios. All the studies we reviewed exhibited significant reductions in their length of stay, with an increased proportion of day 0 discharges. Furthermore, post-operative complications at both 30 days and one year, along with readmission rates, showed no notable differences. The majority of studies also reported a decline in overall hospitalization costs. Patient satisfaction consistently ranged from good to very good.

However, several limitations in our review merit attention: the lack of a universal standard for the ERAS protocol, the limited number of studies addressing ERAS in pelvic floor surgery, the inherent heterogeneity regarding surgical types (sometimes with supplementary procedures included), and the sole reliance on retrospective, single-center studies. Despite the predominantly positive outcomes, these limitations and inherent uncertainties necessitate a cautious interpretation of conclusions. The observed lack of significant differences in operational times and complication rates might further motivate surgeons to incorporate ERAS, potentially paving the way for more extensive future publications.

## Figures and Tables

**Table 4:** Operative duration of surgery, blood loss, length of hospital stay, readmission rates, and hospital costs.

	Operating Time in Minutes			Blood Loss in Milliliters			Length of Hospital Stay in Hours			Readmission Rate after Initial Hospitalization			Total Hospital Costs by Currency of Origin and Euro Equivalent		
	bE	wE	S	bE	wE	S	bE	wE	S	bE	wE	S	bE	wE	S
Carter-Brooks [7]	156	156	0.96 *	64	78	0.35 *	25.9	12.2	<0.01 *	1.5%	6.7%	0.03 *	-	-	-
Evans [8]	160	161	0.48 *	50	50	2 *	28	26	0.01 *	-	-	-	-	-	-
Gong [11]	66	58	0.16 *	90	92	0.26 *	121.4	70.3	<0.01 *	-	-	-	CNY 46,838.65 EUR 6511	CNY 42,793.57 EUR 5948	CNY 4045.08 EUR 562 (*p* < 0.01)
Abrao Trad [12]	-	-	-	-	-	-	-	-	-	6.3%	6.3%	NS	-	-	USD 869 EUR 769
Trowbridge [13]	165	156	0.19 *	102	97	0.72 *	29.9	27.9	0.04 *	3.9%	3.4%	0.84 *	USD 7908 EUR 6997.30	USD 8072 EUR 7142.42	USD 164 EUR 145.11 (*p* = 0.79)
Evans [14]	194	178	0.34 *	50	50	0.60 *	27.1	12.1	<0.01 *	-	-	-	-	-	-
Pan [15]	253	249	8–15 ^†^	124	173	31.2–70.4 ^†^	44.2	33	6.4–16.0 ^†^	-	-	-	-	-	-
Carter-Brooks [16]	-	-	-	-	-	-	25.5	10.5	-	-	-	-	-	-	-
Relph [9]	-	-	-	-	-	-	42.9	23.5	-	0%	0%	NS	GBP 1302 EUR 1548	GBP 1137 EUR 1352	GBP 165 EUR 196
Yoong [10]	90	90	NS	200	175	NS	45.5	22	<0.01 *	0%	4%	0.29 *	GBP 1149 EUR 1366	GBP 1042 EUR 1239	GBP 106 EUR 94

bE: before ERAS; wE: with ERAS; S: significance; *: *p* value; ^†^: 95% confidence interval; NS: not significant; CNY: Yuan; EUR: Euros; USD: US dollars; GBP: Pounds sterling.

**Table 5:** Post-operative complications within 30 days of surgery.

	Complications Taken into Account	Results
	**Within 30 Days of Surgery**	
Carter- Brooks [7]	Intra-operative complications Hospital complications, including hypoxia, chest pain, arrhythmia, hyponatremia, uncontrolled pain, oliguria, nausea/ileus, scar complications Discharge complications, including urinary disorders, scar complications, angina, arrhythmia, nausea/ileus, hematoma, dizziness and ureteral obstruction Unscheduled post-hospitalization consultation Emergency room visit Readmission, including myocardial infarction, chest pain, arrhythmia, hyponatremia, scar complications, nausea/ileus, and ureteral obstruction Surgical re-intervention Urinary tract infection	31.4% before ERAS 35.5% with ERAS *p* = 0.48
Evans [8]	Post-operative complications	5% before and 11% with ERAS, *p* = 0.15
	Emergency room visits	4% before and with ERAS, *p* = 0.99
	Unscheduled consultations	12% before and 7% with ERAS, *p* = 0.29
	Phone calls, median per patient	1 before and with ERAS, *p* = 0.08
Abrao Trad [12]	NA	6.6% before and with ERAS
Trowbridge[13]	Surgical site infection, urinary tract infection, blood transfusion, unplanned surgical re-intervention, pneumonia, pulmonary embolism, unplanned intubation, acute renal failure, cardiac arrest, septic shock, sepsis, death within 30 days, readmission	6.58% before ERAS 8.47% with ERAS *p* = 0.79
Carter- Brooks [16]	Any aberration in the normal recovery	7.6% before and 12.8% with ERAS, *p* = 0.121
Yoong [10]	Emergency room visits	0% before and 12.0% with ERAS, *p* = 0.05
	**One year after surgery**	
Gong [11]	Anterior mesh exposure	9% before and 6% with ERAS, *p* = 0.48
	Posterior mesh exposure	6.4% before and 3.7% with ERAS, *p* = 0.96
	Pain or discomfort during sexual intercourse	34% before and 35% with ERAS, *p* = 0.115 (vs. 30% and 31% before surgery (*p* = 0.19))
	Chronic pelvic pain	5.1% before and 7.3% with ERAS, *p* = 0.57
	De novo urinary incontinence	0% before and with ERAS
	Recurrence of prolapse	0% before and with ERAS

NA: not available; vs.: versus.

## Data Availability

No new data were created or analyzed in this study. Data sharing is not applicable to this article.
